# Treating Methamphetamine-Induced Resistant Psychosis with Clozapine

**DOI:** 10.1155/2014/845145

**Published:** 2014-10-28

**Authors:** Ruohollah Seddigh, Amir-Abbas keshavarz-Akhlaghi, Behnam Shariati

**Affiliations:** Mental Health Research Center, Faculty of Behavioral Sciences and Mental Health, Tehran Institute of Psychiatry, Iran University of Medical Sciences, Tehran, Iran

## Abstract

*Background*. Methamphetamine-induced psychosis (MIP) in Iran has turned into a serious issue in terms of health and treatment, lacking any obvious treatment methods for its resistant cases. *Aims of Case Report*. In the present study, a number of two cases of treatment of MIP with clozapine, which were resistant to the treatment with other antipsychotics, have been reported. Both cases completely responded to the treatment in only 2 weeks and no signs of psychosis relapse were seen in an 8-9 follow-up. *Conclusion*. Because of its particular pharmacologic features, clozapine may be effective in treating MIP.

## 1. Introduction

During recent years, methamphetamine abuse has increased and turned into a serious concern [1–3]. Studies have shown that a chronic use of methamphetamine is accompanied by neurotoxicity, cognitive and psychiatric dysfunctions, and several performance-related troubles [4, 5] imposing huge costs on individuals, families, and society as well [6]. However, what has caused its abuse turning into a serious concern is psychosis induced by it, with it changing into a challenge for the health and treatment system in Iran [7]. Solely, a single experience of substance abuse among the individuals with an underlying psychotic disorder precipitate psychosis in 50–70% of such cases [8]; and among those individuals without any underlying factors, it may cause different types of short-term, long-term, and periodic psychosis or even those types being resistant to treatment with antipsychotic medications [5, 9, 10].

From treatment perspective, although some typical and atypical antipsychotics have been utilized in treating MIP [11], few studies have been conducted regarding resistant psychotic cases. For instance, some reports on the efficacy of electroconvulsive therapy (ECT) among these patients [12]. Moreover, this group of patients seems to have much heterogeneity in their responding to treatment [9].

The present report involves a treatment description of two patients affected by MIP, the symptoms of whom were only cured by taking clozapine. Our searches revealed that no use of clozapine has been reported in treating MIP yet.

## 2. Case Presentation

*The First Patient*. The first patient is a 34-year-old man, single, having primary education, a house painter. He began to consume opium, then heroin at the age of 20, and methamphetamine at the age of 25. During last 9 years, he was hospitalized 12 times due to MIP at psychiatry hospitals. In each hospitalization time, the symptoms were removed by different antipsychotic drugs in only 1-2 weeks; and their relapse again happened due to avoiding taking drugs and starting to consume it as well. In last 6 months, however, despite methamphetamine withdrawal, psychosis continued to remain. In psychiatric examinations, paranoid delusion and commanding auditory hallucinations were reported. Besides, the patient also complained about anxiety and occasional insomnia. He was totally alert answering our questions quite well. However, he was unaware of his psychotic states. In last 6 months, in spite of treatment with the following medications (each prescribed for 4 weeks), the symptoms were not removed: olanzapine: 15 mg daily; risperidone: 6 mg daily; and thiothixene: 15 mg daily.

Due to a failure in recovery from disease, the patient received 6 sessions of ECT. Each session caused seizure lasting averagely for 45 seconds (35–60 seconds) which was observed in the form of muscle contraction in patient's bedside. Due to an occurrence of a long seizure (lasting for 130 seconds) in the 6th session and avoiding it through diazepam intravenous injection, ECT sessions were terminated. During these therapeutic sessions, no change was observed in patient's psychotic state. Eventually, a treatment plan with clozapine (25 mg daily) was started and gradually titrated to 150 mg daily. As a result, psychosis was completely removed in only 2 weeks. In an 8-month follow-up and regarding clozapine consumption to be continued, no relapse in psychotic symptoms was observed. Concerning drug abuse, the patient was in “early full remission” based on DSM-IV-TR criteria despite the fact that he was not in a controlled environment. Thus, he completely withdrew from any substance abuse and returned to his work.

The patient's familial background, regarding axis-I psychiatric disorders, was negative. Moreover, the patient himself had no background of axis-I psychiatric disorders prior to methamphetamine abuse. However, considering the existence of oppositional behaviors in his childhood and adolescence as well as his interview and results of questionnaire of Structured Clinical Interview for DSM-IV-TR Axis II (SCID-II), a diagnosis of antisocial personality disorder was evident for him. According to medical examinations, Complete Blood Count, tests of thyroid, liver and kidney functions, and tests on different hepatitis types and human immunodeficiency virus (HIV) as well as brain MRI were all normal. Besides, after the 6th session of ECT in neurologic counseling, no pathologic point was seen. Thus, it was merely advised to that ECT be terminated. 

*The Second Patient*. The second patient is a 29-year-old man, single, having secondary education. He started substance abuse since he was 15 and has consumed all kinds of illegal drugs in Iran. However, he was affected by psychosis for the first time due to methamphetamine abuse since 6 months ago. In last 3 months, however, despite methamphetamine withdrawal, psychosis continued to remain. In the patient's first visit to psychiatry clinic, persecutory delusion, reference delusion, somatic hallucinations (in a way that the patient felt an outer thing under his hand skin), and olfactory hallucinations (in a way that he felt the smell of opium) as well as anxiety, irritation, and loss of libido were seen. Due to being aggressive, the patient was hospitalized in psychiatric emergency department. Then, clonazepam (6 mg daily) was prescribed for him. In 24 hours, he was seen to be much calmer. However, he was unaware of his psychotic states. In last 6 months, despite treatment with the medications mentioned below (each lasting for 3 weeks), in spite of controlling aggression, psychosis was not removed: olanzapine: 15 mg daily; risperidone: 5 mg daily; quetiapine 600 mg daily; and haloperidol: 10 mg daily. Eventually, all drugs prescribed previously were avoided and clozapine (with a dosage of 100 mg daily), together with clonazepam (2 mg daily), started to be prescribed. One week after clozapine prescription, clonazepam was discontinued. In a 9-month follow-up and with the continuance of clozapine consumption, no signs of psychosis relapse were seen. Concerning drug abuse, he had no other abuse experience; and the patient returned to his work. So, he was in “early full remission” based on DSM-IV-TR criteria despite the fact that he was not in a controlled environment.

The patient's familial and personal backgrounds, regarding axis-I psychiatric disorders were negative. However, a diagnosis of antisocial personality disorder was evident for him. Moreover, similar to the previous patient, all paraclinical examinations proved to be negative.

## 3. Discussion

Although MIP alleviates after a short period of time among numerous patients, it may last for several months in spite of stopping its abuse and taking antipsychotics as well; thus, pharmacologic therapy is indicated [9]. In many of studies, atypical antipsychotics have been prescribed in order to treat MIP, and the efficacy of such drugs as risperidone [13], olanzapine [14], quetiapine [15, 16], and aripiprazole [17] in treating it to some extent has been demonstrated. However, in the state of drug-resistant psychosis, there is no reliable evidence. For example one study has reported the positive influence of ECT in these cases [12]. In the present study, clozapine was prescribed due to the patients' not responding to these treatment methods. Both patients continued to suffer from psychosis even after receiving at least 3 periods of treatment with nonclozapine antipsychotics. However, their symptoms were completely removed in only a short time period since taking clozapine.

From a neuroanatomical perspective, methamphetamine abuse with a low dosage causes damage to serotonergic pathways in areas of frontal cortex and hippocampus; and its high dosage abuse leads to some damage to striatum and parietal cortex [4, 18]. Considering neurotransmitters, methamphetamine leads to the release of high amounts of dopamine, norepinephrine, and serotonin in the brain, especially in striatum and its related areas [19]. But, in the case of its long-term abuse, it decreases not only the density of the above-mentioned neurotransmitters (especially dopamine) in the brain due to its neurotoxic effects [18] but also the number of D_2_ dopamine and dopamine transporter receptors [20]. Such effects may continue to occur even after a long withdrawal from abuse. On the other hand, a long-term abuse of methamphetamine may cause noradrenergic hyperactivity, individuals' sensitivity to stress, and psychosis relapse [21]. Moreover, studies have shown that, in patients affected by a spontaneous relapse of psychosis after a long-term withdrawal, when they experience flashbacks, the norepinephrine and 3-methoxytyramine (the major metabolite of dopamine) blood levels are high in their blood [22]. The above profile represents the major excitatory effects of methamphetamine.

On the other hand, among antipsychotics, clozapine possesses unique features, compared to other antipsychotics. Clozapine has a high affinity towards 5-HT_2_ receptor blockade. This receptor has huge stimulating effects in the brain and is blocked to a high extent as the patient takes clozapine. Moreover, clozapine is a strong blocker of 5-HT_3_ as well as *α*_1_ and *α*_2_ adrenergic receptors [23]. Regarding this profile, clozapine seems to overcome adrenergic and serotonergic arousal seen in methamphetamine abuse. Thus, it may be considered as a treatment for MIP. Among other antipsychotics, such effects are also observed in aripiprazole [24]; however, according to very limited studies available, this drug just reduces the severity of psychosis in MIP [17]. Due to its unavailability, aripiprazole was not prescribed for the patients in this study.

Among clozapine effects, one other unique effect is its lower infinity towards D_2_ receptor blocks, compared to other antipsychotics in general, and toward many of D_2_ receptor blocks in mesolimbic areas, compared to those receptors in striatum areas [23]. This means that, considering neuropathology, mesolimbic areas may receive more importance than striatum areas do among those patients affected by MIP, with this demanding more future research on it.

The other important point made about the two patients in this report is their being in early full remission in an 8-9 follow-up. This means that clozapine may expert positive effect on some aspects related to methamphetamine abuse such as craving, those mood symptoms accompanied by psychosis, anxiety, and aggression. However, more controlled research needs to be done in an attempt to confirm this.

## Abbreviations

MIP: Methamphetamine-induced psychosis
ECT: Electroconvulsive therapy.
